# Building Potent Chimeric Antigen Receptor T Cells With CRISPR Genome Editing

**DOI:** 10.3389/fimmu.2019.00456

**Published:** 2019-03-19

**Authors:** Jie Liu, Guangyu Zhou, Li Zhang, Qi Zhao

**Affiliations:** ^1^Cancer Centre, Faculty of Health Sciences, University of Macau, Macau, China; ^2^Institute of Translational Medicine, Faculty of Health Sciences, University of Macau, Macau, China; ^3^Department of Molecular and Cellular Oncology, The University of Texas MD Anderson Cancer Center, Houston, TX, United States

**Keywords:** chimeric antigen receptor, CRISPR, gene editing, immunotherapy, cancer, CAR T

## Abstract

Chimeric antigen receptor (CAR) T cells have shown great promise in the treatment of hematological and solid malignancies. However, despite the success of this field, there remain some major challenges, including accelerated T cell exhaustion, potential toxicities, and insertional oncogenesis. To overcome these limitations, recent advances in CRISPR technology have enabled targetable interventions of endogenous genes in human CAR T cells. These CRISPR genome editing approaches have unleashed the therapeutic potential of CAR T cell therapy. Here, we summarize the potential benefits, safety concerns, and difficulties in the generation of gene-edited CAR T cells using CRISPR technology.

## Introduction to Chimeric Antigen Receptor T Cell Therapy

Major histocompatibility complex (MHC) molecules play key roles in the surveillance of aberrant proteins of tumor cells. T cell receptors (TCRs) on the surface of T lymphocytes recognize antigenic peptide fragments derived from these aberrant proteins in complex with MHCs (1, 2). The expression of MHC/peptide complexes constitutively occurs on all nucleated cells. Tumor-specific MHC/peptide complexes are considered ideal targets for T cell-based immunotherapies. Diverse strategies have been developed to induce T cell immunity against these tumor epitopes, including cancer vaccination (3), adoptive T cell transfer (4), and TCR engineering (5). In cancer patients, however, tumor cells can effectively escape adoptive immunity via regulatory mechanisms, such as downregulation of MHCs or mutation. Because the presence of relatively fewer MHC molecules on the tumor cell surface limits naive TCR recognition, T cells fail to respond and trigger cascades of immune activation (6).

**Figure 1:**
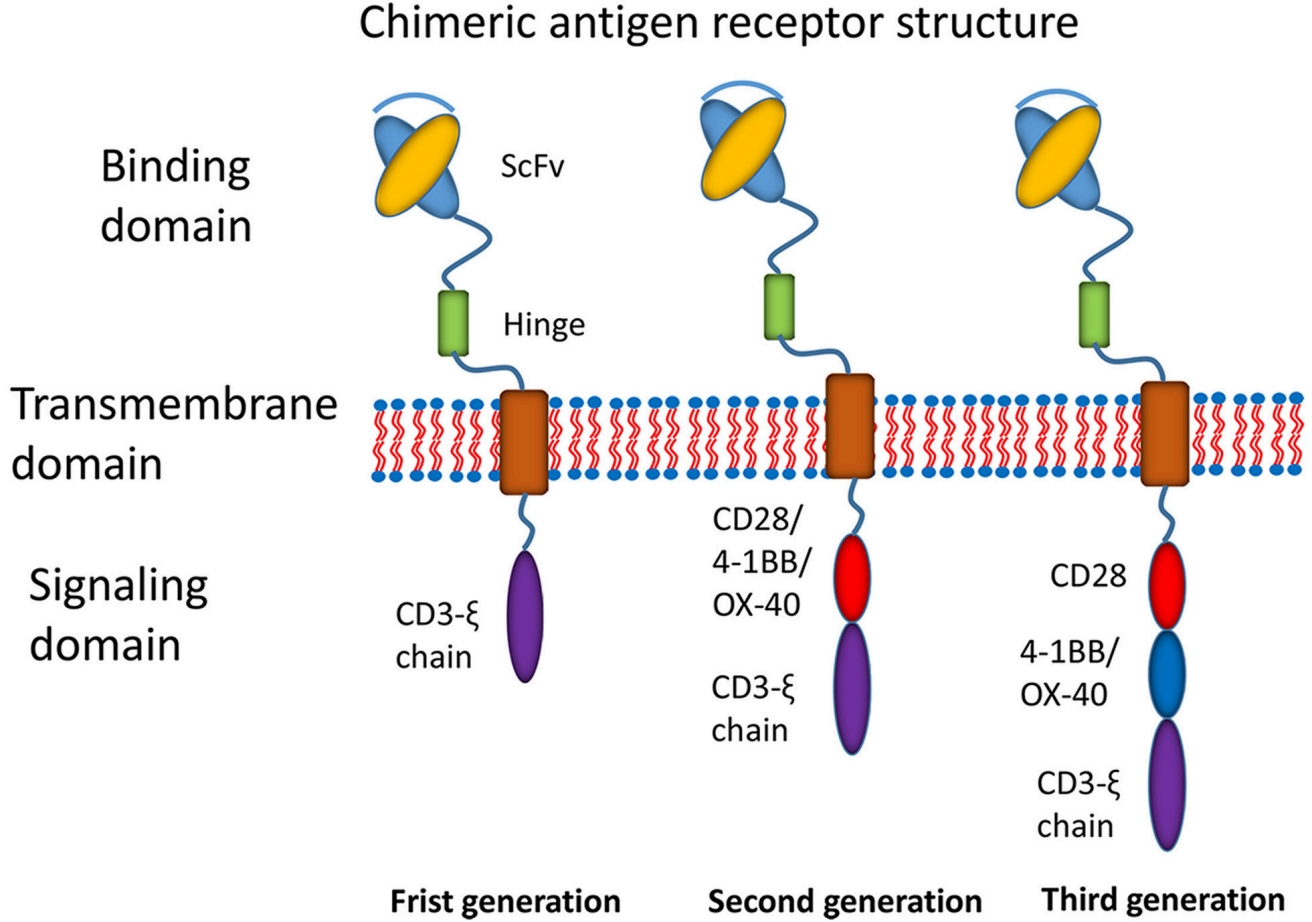
Main structures of chimeric antigen receptors. Three generations of CAR structures. In the first generation of CARs, the binding domain (single chain antibody fragment, scFv) is linked to the signaling domain (CD3ζ) via the transmembrane domain. In the second generation of CARs, the costimulatory molecule (CM1, such as CD28 4-1BB or OX-40) is introduced with the signaling domain (CD3ζ). In the third generation of CARs, the additional costimulatory molecule (CM2) is included.

Recently, the most promising development has been the use of chimeric antigen receptor (CAR) T cell immunotherapy (7). CAR T cell immunotherapy has emerged as a leading curative strategy in the treatment of relapsed hematological malignancies. CAR T cell therapy is based on the immune effect of T cell activation and the principle of transformation through the genetic engineering of T cells. A typical CAR construct comprises a binding domain (single chain antibody fragment, scFv), a transmembrane domain and intracellular signaling domains capable of activating T cells (Figure 1). CARs allow the T cells to be activated independently of MHC. Donor-derived T cells are modified to express multivalent CARs on the cell surface that are responsible for recognizing the tumor-associated antigen (TAA) of tumor cells. Thus, T cells are activated via intracellular signal transduction. CAR designs differ not only in their signaling domains but also in their functional properties. The CAR structures have progressed since the first generation was described in 1989 (8). The first generation of CARs was designed as an scFv linked to the CD3ζ intracellular signaling domain of the TCR through a hinge and a transmembrane domain. Although the CD3ζ signaling domain can trigger activation of T cells, this pattern most likely results in T cell anergy, attenuating T cell activation. Therefore, the first generation of CARs exhibited limited responses in clinical trials (7). To address this limitation, a costimulatory molecule, such as CD28, OX40, or 4-1BB, was incorporated into the intracellular domain for the second generation of CARs. The additional costimulatory domain in the second generation of CARs strikingly improved T-cell proliferation and persistence. To optimize T-cell efficacy, the third generation of CARs has been developed by introducing two costimulatory domains into the CAR structure. Although dual costimulatory domains can enhance the activation and proliferation of T cells, the abundance of cytokines remains to be considered.

The CAR T cell approach has provided great advances in the treatment of hematological malignancies. Anti-CD19 CAR T cells have significantly advanced the therapy of human hematological malignancies and were shown to achieve a 90% complete response rate in acute lymphoblastic leukemia (ALL) (9). Tisagenlecleucel, the first anti-CD19 CAR T cell therapy, was approved by the US Food and Drug Administration (US FDA) for the treatment of children and adults with advanced leukemia in 2017 (10, 11). As 2017 ended, there were hundreds of ongoing CAR T cell trials for the treatment of hematologic and solid tumor malignancies (12).

## Possible Side Effects of Chimeric Antigen Receptor T Cell Therapy

Although most patients infused with CAR T cells show mild or moderate side effects, potentially severe side effects are still challenging. The prominent toxicities include cytokine release syndrome (CRS), insertional oncogenesis, and neurologic toxicity (13, 14).

### Cytokine Release Syndrome

CRS is an unintended side effect due to overactivation of the host immune system. Severe CRS was observed in some patients who received infusion of CAR T cells (15). An abundance of cytokines is released by either the infused CAR T cells or other polarized immune cells. Several clinical studies indicated that 19–43% of patients exhibited CRS when they were treated with anti-CD19 CAR T cells for relapsed/refractory ALL (13, 16). Clinical features of CRS include high fever, muscle pain, malaise, unstable hypotension, fatigue, ang capillary leakage (17). A wide variety of cytokines can be elevated in the serum of patients. Dramatic elevations of inflammatory cytokines, such as INF-γ, IL-2, IL-6, and IL-10, are observed in CRS (18). Occasionally, neurologic toxicity can be associated with anti-CD19 CAR T cell therapy, probably due to the elevated levels of cytokines (16). The use of the anti-IL-6 receptor antibody tocilizumab was demonstrated to exert curative effects for serious cases of CRS in all patients with a high proliferation of CAR T cells (19).

### Insertional Oncogenesis

Continuous CAR expression in T cells relies primarily on the delivery of the CAR gene by integrated gamma retroviral (RV) or lentiviral (LV) vectors. The advantages of both systems are high gene-transfer efficiency and stable expression of the CARs. Although both RV and LV vectors have been shown to be safe in intensive biosafety testing, this safety issue remains a concern. LV- or RV-mediated random and uncontrollable integration in the genome are unpredictable (20). Uncontrollable insertions of CAR genes lead to potential oncogenesis, variegated transgene expression, and transcriptional silencing (21). This possibility poses an oncogenic risk for RV/LV-engineered T cells (22). Although RV-driven oncogenesis has not yet been reported in CAR T cell therapy, this phenomenon was observed in clinical trials of hematopoietic stem cell transplantation (23). Additionally, random integration into the genome causes substantial variations in CAR expression levels in a batch of CAR T cells because of the different copy numbers per cell.

### Graft-vs.-Host Disease

With the gradual initiation of clinical trials, autologous CAR T cells have shown some disadvantages. In infants or adults who are receiving chemotherapy or radiotherapy, it is difficult to harvest sufficient lymphocytes for CAR T cell manufacture. Thus, the quality of CAR T cells for each patient is uncontrollable and unpredictable. The use of allogeneic CAR T cells has become a solution for these problems. Allogeneic CAR T cells can be expanded *ex vivo* on a large scale and can be reserved to treat multiple patients (24). The concerned with allogeneic infusion is graft-vs.-host disease (GVHD) between the donor cells and recipients. The repertoire of TCRs and MHCs expressed on allogeneic CAR T cells may potentially induce GVHD in recipients who receive donor CAR T cells (25). A study showed that allogeneic anti-CD19-CAR T cells had clinical benefits for relapsed hematologic malignancies (26). No obvious GVHD was observed in these recipients.

## Generation of Potent CAR T Cells With CRISPR Technology

**Figure 2:**
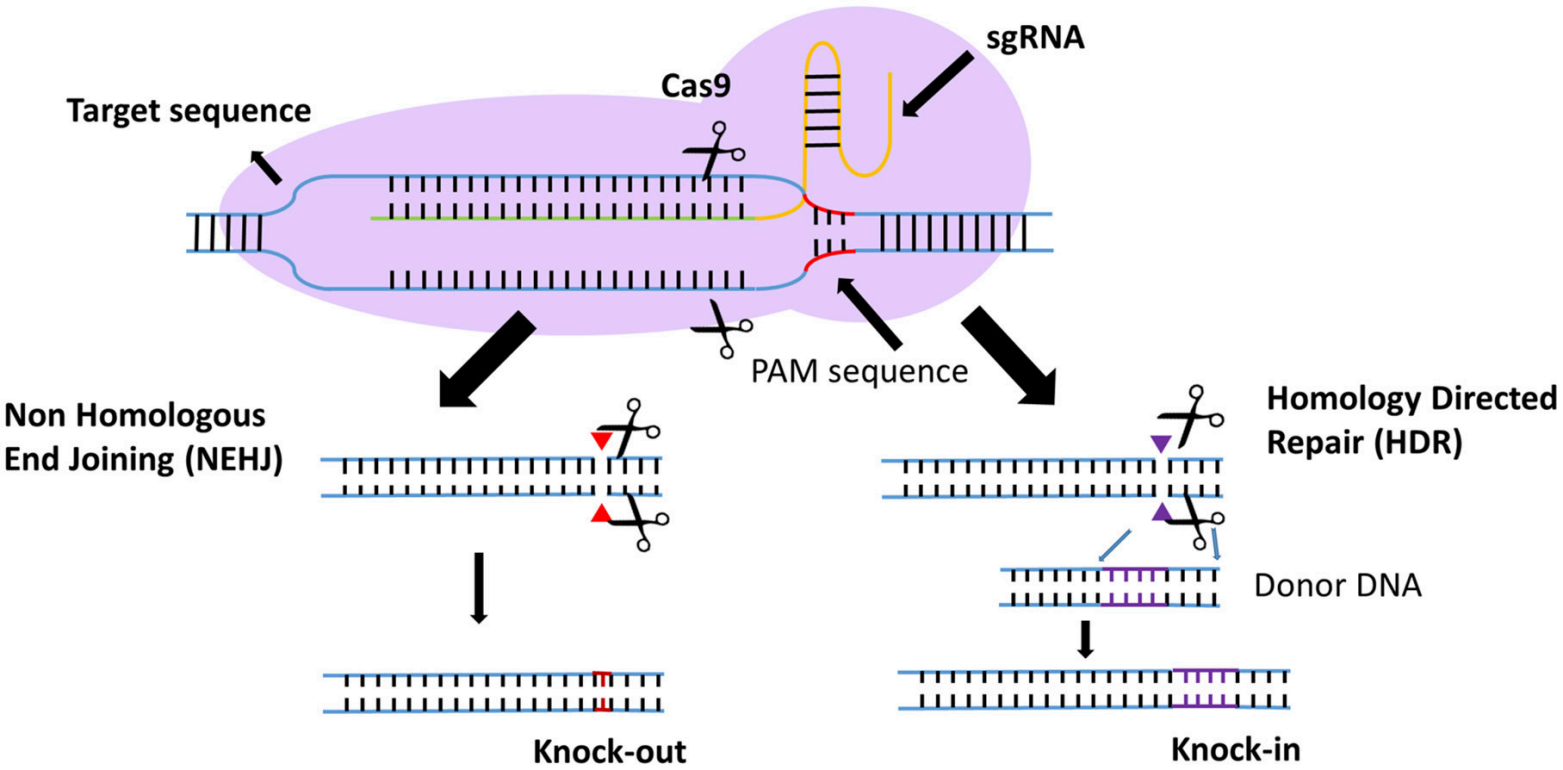
Introduction to the CRISPR gene-editing system. Guided by sgRNAs, the CRISPR-Cas9 nuclease can target short DNA sequences. The PAM specifically creates a sgRNA–target DNA heteroduplex and generates double-strand breaks. Then, the DNA double-strand breaks are repaired by non-homologous end-joining (NHEJ) or homology-directed repair (HDR). In the NHEJ pathway, indels lead to nucleotide deletions or insertions. In the HDR pathway, accessory factors can facilitate genome recombination through the two homology arms, resulting in the knock-in of a gene of interest.

Efforts to enhance the efficacy of CAR T cell therapy have been undertaken, including the selection of extracellular receptors (27), optimization of intracellular costimulatory molecules (28), combination with cytokines(29), and improvement of “on-target/off-tumor” toxicity (30). Effective gene-editing technologies have emerged as tools for cell engineering (31). The properties of three gene-editing tools, including CRISPR, zinc-finger nucleases (ZFNs), and transcription activator-like effector nucleases (TALENs), are summarized in Table 1. The use of CRISPR in genome editing is highly efficient and enables a simple and efficient way to multiplex the processing of T cells (32, 33). Both ZFNs and TALENs have also been adopted to modify T cells for clinical applications (34, 35). However, the recognition of the targetable DNA sequences with ZFNs and TALENs in T cells remains complicated and tedious, resulting in a low gene-editing efficiency. The simultaneous multiplexed genetic manipulations of these techniques are challenging (36). CRISPR/Cas9 systems have been used for the knock-out and knock-in of sequences in mammalian genome editing (Figure 2). In principle, a deletion or insertion at a target gene is introduced by a small RNA (sgRNA)-guided Cas9 nuclease that induces a double-stranded DNA break, which is subsequently repaired by non-homologous end joining (NHEJ) (37). Nucleotide insertions or deletions result in non-sense mutations and loss of gene function. In comparison to NHEJ, a relatively large gene sequence can be delivered to a precise locus in the genome through homology directed repair (HDR) after double-stranded DNA is cleaved by sgRNAs (38–40). The HDR process enables precisely targeted nucleotide replacements at the defined site of interest. Currently, several strategies based on CRISPR are being applied to develop next-generation CAR T cells by multiplexed genome editing (41–43). Such approaches include the knockout of endogenous genes (such as TCRs, MHCs, or self-antigens) to build allogeneic universal CAR T cells (41, 44, 45), the disruption of inhibitory receptors (such as CTLA-4, PD-1, or LAG-3) (44, 46, 47), and the integration of the CAR cassette into the endogenous TCR α constant locus (TRAC) (48, 49) or the C-C chemokine receptor type 5 (CCR5) locus (32) (Table 2).

**Table 1:** Comparison of ZFN, TALEN, and CRISPR.

**Property**	**ZFN**	**TALEN**	**CRISPR**
Anchor site	18–36 nt	30–36 nt	23 nt
Off-target	Low	low	High
Complication	High	High	Low
Efficiency	Relatively low	Relatively low	High
Multiplex	Low	Low	High
Methylation sensitivity	High	High	Low
Mechanism of action	Zinc finger nuclease for DNA recognition and cleavage	transcription activator-like effector nuclease recognition and DNA cleavage	Guide RNA for DNA recognition and Cas9 endonuclease for cleavage

**Table 2:** Summary of the CAR-T cells modified with gene editing.

**CAR**	**Gene-editing method**	**Targeted gene**	**Gene editing efficiency (%)**	**Malignancy**	**Reference**
**KNOCK-OUT**					
CD19 scFv/4-1BB/CD3ζ	Cas9 RNP electroporation	TRAC	85	B cell acute lymphoblastic leukemia	(41, 42)
		β2M	100		
		PD-1	64.7		
CD19 scFv/4-1BB/CD3ζ	Cas9 RNP electroporation	TRAC	81.7	B cell acute lymphoblastic leukemia	(45)
		TRBC	49.3		
		β2M	79.9		
CD7 scFv/CD28/4-1BB//CD3ζ	Cas9 RNP electroporation	CD7	89.14	T cell acute lymphoblastic leukemia	(44, 52)
EBV-LMP2A CTL	Cas9 plasmid electroporation	PD-1	47.4	Epstein-Barr virus-associated gastric cancer	(46)
CD19 scFv/4-1BB/CD3ζ	Cas9 RNP electroporation	LAG-3	45–70	B cell acute lymphoblastic leukemia	(47)
**KNOCK-IN**					
CD19 scFv/4-1BB/CD3ζ	Cas9 RNP electroporation and transfection with AAV6 encoding CAR	TRAC exon 1	50	B cell lymphoma	(48)
CD19 scFv/CD28/CD3ζ	Cas9 RNP electroporation and transfection with AAV encoding CAR	TRAC exon1	40	Adult B acute lymphoblastic leukemia	(49)

### Universal CAR T Cells

Although autologous CAR T cells against B cell malignancies have shown promising results, some clinical studies demonstrated that for some patients, autologous T cells could not be manufactured due to poor lymphocyte counts or low T cell quality and quantity (50). Especially for some patients in infancy, sufficient peripheral blood mononuclear cells (PBMC) cannot be harvested to support T cell manufacture *ex vivo*. These limitations can be circumvented by utilizing allogeneic T cells. Endogenous TCRs that allogeneic T cells express can recognize the alloantigen of the recipient, resulting in major graft-vs.-host disease (GVHD). Before these allogeneic T cells can be widely used clinically, the issue of GVHD must be resolved (45). Universal allogeneic CAR-T cells are ideal because their manufacture and quality may be more easily controlled and GVHD may be avoided. Several groups have generated allogeneic universal anti-CD19 CAR T cells by deleting multiple genes, such as TRAC, β2M, and MHC, using CRISPR methods (41, 42). Meanwhile, ongoing clinical trials have shown that a suicide gene in the CAR construct can also be used to avoid GVHD after allogeneic CAR T cell injection (25). These results suggest that CAR T cells that utilize multiplexed gene editing generate CAR T cells that are as potent as non-gene-edited T cells.

Until now, most successful CAR T cell therapies have been applied to B cell malignances. For T cell malignances, patients would receive allogeneic T cells rather than autologous CAR T cells. Genomic editing of some antigens, which recognize those “non-self” molecules and are attacked by the host immune system, can broaden the application of CAR T cells. DiPersio et al. reported that fratricide-resistant “off-the-shelf” universal T cells generated with CRISPR gene editing were used for treatment of T-cell malignancies (44). CD7 is a molecule commonly expressed in T lymphocytes. To avoid self-elimination, the CD7 target antigen against malignancies, which is recognized by anti-CD7-CARs, is deleted on CAR T cells (51, 52).

### Resistance to PD-1 Inhibition

It is widely accepted that the existence of immune checkpoints (such as PD-1, CTLA-4, and LAG-3) can attenuate the activation of CAR T cells and accelerate T cell exhaustion. PD-1 is a primary inhibitory molecule in T cell transduction (53, 54). The PD-1/PD-L1 pathway plays an important role in the regulation of T cell activation and differentiation (55). High expression of PD-1 accelerates T cell tolerance and exhaustion (56–59). Increasing evidence indicates that blocking the PD-1/PD-L1 axis could partially restore the function of exhausted T cells (54, 60). A recent clinical study demonstrated that treatment with anti-CD19 CAR T cells in combination with an anti-PD-1 antibody was effective in patients with relapsed chronic lymphocytic leukemia (CLL) (61). This anti-PD-1 antibody treatment revives the antitumor response of anti-CD19 CAR T cells in patients who fail to respond to CAR T cell treatment (62). In other cases, unanticipated autoimmune responses are associated with anti-PD-1 checkpoint inhibitors (63). Therefore, ablation of PD-1 with gene editing by CRISPR/Cas9 is an alternative to enhance the antitumor response of CAR T cells in anti-CD19 CAR T cell therapy (41, 42). Ren et al. suggested that depletion of PD-1 genes in anti-prostate stem cell antigen (PSCA) CAR T cells with a Cas9/RNP method significantly enhanced T cell immunity *in vivo* (42). A significant antitumor response was observed after PD-1 was disrupted by genome editing. Controversially, a study indicated that T-cells without PD-1 were susceptible to exhaustion and lacked long-term durability (64). In regard to other checkpoint targets, no obvious improvement was confirmed when LAG-3 genes were deleted in CAR-T cells using CRISPR/Cas9 (47). Nevertheless, these studies still support the promise of checkpoint inhibition in CAR T cell therapy.

### Targeted Integration of CARs

Recently, effective homologous recombination was shown to promote the site-specific integration of large transgenes in the T cell genome (65). In this method, after the DNA of the target gene is cleaved using Cas9 RNPs, a gene of interest is subsequently delivered to the cleavage site using adeno-associated viruses (AAVs). Site-specific transgene integration is achieved by HDR. An anti-CD19 CAR gene has been successfully integrated into the TRAC locus using the combined action of Cas9/RNP and AAV donor vectors (49). Targeting the CAR gene to the TRAC locus not only results in uniform CAR expression but also delays effector T-cell differentiation and exhaustion. Moreover, the insertion of a CAR transgene into a defined location avoids the risk of insertional oncogenesis and places CAR expression under the control of endogenous regulatory elements.

## Safety Concerns of CRISPR Gene-Edited CAR-T Cell Therapy

To date, although many limitations of conventional CAR T cells have been addressed with CRISPR gene editing, safety issues must be addressed before these gene-edited cells start to move into clinic. Multiple elements, such as off-target effects, Cas9 activity, target site selection, and sgRNA design, and delivery methods, can determine the efficiency and safety of the CRISPR/Cas9 system.

The first concern of CRISPR gene editing is off-target effects (66). These off-target effects might be beneficial to bacteria and archaea (67). However, several recent studies have reported unintentional CRISPR/Cas9-induced large genomic deletions or gene inversions in various species, including mouse, *C. elegans*, and rabbit (68–70). For human therapies, clinical safety is particularly important. Several recent studies have reported off-target effects of CRISPR in T-cells. Off-target effects introduce random mutations, thus impacting tumor-suppressor genes or activating oncogenes. Off-target effects were also observed when the *TRAC* or *TRBC* locus of CAR-T cells was inserted with CRISPR/Cas9 electroporation (42). A controversial study indicated that CRISPR gene editing could cause hundreds of unintended mutations in the genome when whole-genome sequencing was performed on a CRISPR–Cas9-edited mouse (68). Notably, another study showed that CRISPR/Cas9 genome editing resulted in a p53-mediated DNA damage response in human retinal pigment epithelial cells (71). p53 activation may lead to chromosomal rearrangements and other tumorigenic mutations in cells. Although the outcome of CRISPR-induced p53 activation is unconfirmed, it seems to decrease the gene editing efficiency. Therefore, the off-target issues must be considered in the future development of CRISPR/Cas9-edited CAR T cells. Off-target assays during CRISPR target selection may be performed to manage the safety risk of clinical CAR T trials.

Another safety concern is that unpredicted translocations may occur between double-strand breaks when multiple genes are edited (72). Although such events are rare in T cells, transformation analysis should still be performed to ensure the safety of gene-edited CAR-T therapy. In addition to the safety risk of translocations, altered functions of gene-edited CAR-T cells most likely would cause adverse effects in patients. For example, CRISPR gene disruption in CAR T cells can cause unintended innate immune responses (73).

## Perspectives of CRISPR Gene-Edited CAR-T Cell Therapy

In recent, many antitumor approaches have been developed, including target small molecules (74, 75), antibody drugs (76–84), immune cell therapy (85). Among them, CAR T cell therapy aims to treat cancer through the use of the patient's immune system. This type of therapy has many advantages, such as low toxicity and a long duration (86). However, CAR T cell therapy appears to be effective only in a limited portion of patients with hematological malignancies. CRISPR is a cutting-edge technique that can be used to generate CAR T cells with enhanced potency and safety. Although the clinical use of allogeneic donor CAR cells has been recently reported, their use is highly dependent upon either rigorous patient selection or T cell selection (25). Potential GVHD still limits the wide application of allogeneic CAR cells. Taking advantage of CRISPR, the risk of GVHD may be minimized through the deletion of endogenous TCR and MHC molecules. The additional disruption of PD-1 is believed to optimize the antitumor activities of CAR-T cells through the regulation of T-cell functions (32). The safety of gene-edited CAR T cells is the primarily concern because of notorious off-target effects. To minimize the safety risk of off-target effects, careful selection of the target site combined with prior off-target assays will be required during target site selection of CAR T cells. Although skeptics question whether CRISPR gene-edited T cell therapy is safe and ready for the clinical stage, the first CRISPR gene-editing trial using autologous T cells was initiated to treat patients with melanoma, synovial sarcoma, and multiple myeloma in 2016 (87). These potent T cells have shown merits in preclinical studies. The long-term safety profile of gene-edited CAR-T cells should be further examined in the clinic.

## Author Contributions

JL, GZ, and LZ wrote part of the manuscript; QZ wrote the manuscript.

### Conflict of Interest Statement

The authors declare that the research was conducted in the absence of any commercial or financial relationships that could be construed as a potential conflict of interest.
